# Demographic and professional profile of Brazilian women in vascular surgery: final results

**DOI:** 10.1590/1677-5449.210062

**Published:** 2021-08-13

**Authors:** Fernanda Costa Sampaio Silva, Monique Magnavita Borba da Fonseca Cerqueira, Magno Conceição das Mercês, Flávia Magalhães Silveira Magella, Bárbara Beatriz Couto Ruivo, Marita von Rautenfeld, Roque Aras

**Affiliations:** 1 Universidade Federal da Bahia – UFBA, Salvador, BA, Brasil.; 2 Universidade do Estado da Bahia – UNEB, Salvador, BA, Brasil.; 3 Clínica Magella, São Paulo, SP, Brasil.; 4 Pierre and Marie Curie University, Paris, France.; 5 Hospital São Camilo, São Paulo, SP, Brasil.

**Keywords:** medical education, sexism, leadership, surgical specialties, surveys and questionnaires, educação médica, sexismo, liderança, especialidades cirúrgicas, inquéritos e questionários

## Abstract

**Background:**

Gender diversity in health teams is associated with better productivity. As women’s participation in surgery has been growing, it is important to improve knowledge about the elements that guide their professional development.

**Objectives:**

The aim of this study was to outline the demographic and professional features of female vascular surgeons in Brazil.

**Methods:**

A cross-sectional study was designed, in which a questionnaire was made available online for 60 days. Invitations to participate were distributed by institutional e-mail sent from the Brazilian Society of Angiology and Vascular Surgery (SBACV) to associate women surgeons. Results are presented as numbers and percentages. Odds ratios and chi-square tests were used for analysis.

**Results:**

From a total of 810 invitations sent out, 281 questionnaires were completed. The most prevalent age groups were 25-35 years (n = 115) and 36-45 years (n = 114). Among those who worked exclusively in the private sector, 79.8% had at least one board certification (OR: 0.76, 95% CI: 0.65-0.89; p = 0.001). Regarding workload distribution, 64.4% and 34.2% reported that they spend more time in the clinic and hospital, respectively. Respondents with more years of experience reported a predominance of office practice (p = 0.002). Although 67.3% (n = 189) had published scientific papers, 68% (n = 191) had never held leadership roles.

**Conclusions:**

The study respondents consisted of highly qualified women surgeons with respect to training, certification, and scientific engagement, but they remain underrepresented in professional management positions. Surgical societies and health institutions should act to promote inclusive and diverse leadership.

## Resumo

**Contexto:**

A diversidade de gênero em equipes médicas está associada a uma melhor produtividade. Com o aumento da participação feminina na cirurgia, é importante conhecer melhor os elementos que orientam o desenvolvimento dessas profissionais.

**Objetivos:**

Delinear as características demográficas e profissionais das cirurgiãs vasculares no Brasil.

**Métodos:**

Estudo de caráter transversal, em que foi disponibilizado um questionário on-line por 60 dias. O convite foi enviado pelo e-mail institucional da Sociedade Brasileira de Angiologia e Cirurgia Vascular (SBACV) às cirurgiãs associadas. Os resultados são apresentados como números e porcentagens. Os testes de *odds ratio* (OR) e do qui-quadrado foram utilizados para a análise.

**Resultados:**

De 810 convites enviados, 281 questionários foram respondidos. Os grupos etários predominantes foram 25-35 anos (n = 115) e 36-45 anos (n = 114). Entre as que trabalhavam exclusivamente no setor privado, 79,8% possuíam ao menos um Título de Especialista (OR: 0,76; intervalo de confiança de 95% 0,65-0,89; p = 0,001). Em relação à distribuição da carga horária, 64,4% e 34,2% relataram que passavam mais tempo no ambulatório e no hospital, respectivamente. Entre as com mais anos de experiência, houve predomínio da prática em consultório (p = 0,002). Embora 67,3% (n = 189) tenham publicado artigos científicos, 68% (n = 191) nunca ocuparam cargos de liderança.

**Conclusões:**

As participantes do estudo consistiram em cirurgiãs altamente qualificadas em relação a treinamento, certificação e engajamento científico. No entanto, permanecem pouco representadas em cargos de gestão profissional. As sociedades cirúrgicas e as instituições de saúde devem agir para promover uma liderança inclusiva e diversa.

## INTRODUCTION

In the United States (US), women currently account for 34% of practicing doctors, approximately 46% of doctors in training, and more than half of medical students.^1^ In Brazil, following a global trend, women are already a majority among recent medical school graduates and doctors under the age of 35 years, accounting for approximately 57.4% of the group aged ≤29 years and 53.7% of the group aged between 30 and 34 years.^2^ Nevertheless, with regard to their representation in the surgical specialty, women are still a minority.^3^^,^^4^ Among Brazilian vascular surgeons, women constituted 22.9% of the workforce, which equated to 4,301 professionals by 2018, according to data from the Federal Medical Council.^2^ Some authors believe that one of the factors responsible for this scenario is presence of organizational barriers,^5^ which impose rigid and inflexible work expectations on surgeons, making the profession seemingly incompatible with family demands.^6^^,^^7^

Another aspect that should be discussed is the influence of mentors during medical training, suggesting that progression of women in the field of surgery would be leveraged if students had the support and guidance of women leaders.^8^ Furthermore, the gender discrimination which remains within the field of surgery cannot be overlooked because it perpetuates the obsolete concept that women are less technically fit and thereby limits women’s opportunities for education, clinical roles, research, and leadership.^6^^,^^9^^,^^10^ Fortunately, during the last 5 years, there has been a noticeable increase in women’s representation in the highest positions of international surgical societies.^11^ However, there is still a discrepancy between the number of women surgeons and their representation in prominent positions.^12^ This is particularly concerning, considering that these promotions depend on the recognition and legitimization of their peers.

It is important to address barriers facing women who are interested in becoming surgeons to avoid a continuation of the current trends.^5^ Most studies, however, have focused on aspects directly related to training.^1^^,^^7^ There are few examples of studies that have addressed professional elements particular to women surgeons after completion of medical residency and their participation in the workforce.^11^^,^^12^

Based on the current situation, the objective of the present study was to describe the demographic and professional profile of Brazilian women vascular surgeons.

## METHODS

This was a cross-sectional descriptive study. To foster discussion on the topic and evaluate the target audience’s receptivity to the research, a pilot study was conducted in June 2017, in which a 15-question electronic questionnaire was prepared and made available through the portal survio.com for 30 days. Invitations to participate in the study were distributed using social networks of women vascular surgeons in Brazil. Consequently, 101 questionnaires were completed, and results were published in the *Jornal Vascular Brasileiro* in June 2018.^12^ After publication of the preliminary study, the present study was designed based on the same questionnaire. An electronic informed consent form was required, comprising item 1 on the questionnaire. Item 2 was defined as the research subject code, which included the participants’ name and registration number with the Regional Council of Medicine to ensure that there were no duplicate responses or questionnaires from people outside the study target population. Items 3 to 16 of the survey were about demographics, training, integration into the labor market, and scientific and leadership involvement. The survey link was again made available through survio.com, remaining online from July 16, 2018, to September 15, 2018. Participants were invited once via the Brazilian Society of Angiology and Vascular Surgery (SBACV) institutional email account. The population was estimated at 810 women vascular surgeons linked to the abovementioned society, according to the institution's database. Assuming a confidence level of 95% and a maximum margin of error of 4% and using the OpenEpi^13^ Version 3.01 open source calculator, the target sample size calculated by proportion for this study equaled 261 questionnaires. The study received institutional unpaid support from SBACV and was approved by the Research Ethics Committee of the lead author’s institution under protocol CAAE:89316318.6.0000.5606 and Consubstantiated Opinion number 3,146,091. In March 2020, an editorial^14^ for debate on this topic was published by the same authors in the European Journal for Vascular and Endovascular Surgery and detailed results are now published in the present manuscript.

### Statistical analysis

Questionnaires with a “No” answer to item 1 requesting informed consent were excluded from analyses. SPSS for Windows version 25.0 (IBM Corp., Armonk, NY, USA) was used for tabulation and analysis. Results are presented as numbers and percentages. The odds ratio chi-square test was used for categorical variables and the non-parametric Pearson’s chi-square test was employed for continuous quantitative variables. For all analyses, a value of p ≤ 0.05 was established.

## RESULTS

From a total of 810 invitations sent out, 287 questionnaires were answered. All participants marked “Yes” in response to the informed consent question. Due to inconsistencies in the registration data collected for item 2, 6 questionnaires were excluded, resulting in a total of 281 valid responses. The data collected in the pilot study were not included in the present study. The full questionnaire is shown in Supplemental Table 1.

### Demographic data

All demographic data are summarized in Supplemental Table 2. In total, 280 participants identified themselves as Brazilian and 1 respondent was of foreign nationality (American), according to responses from Question 3. Regionally, most women surgeons were from the Southeast (54.8%), followed by the Northeast (18.1%), South (13.2%), Midwest (10%), and North (2.8%) regions of Brazil, as determined by answers to Question 4 (“Which state do you practice in?”). Of these, 3 women (1.1%) reported working simultaneously in two regions, Southeast/South, Southeast/Northeast, and Southeast/Midwest (Supplemental Figure 1), since this question allowed for more than one answer per participant. This is because there is no legal impediment to Brazilian physicians working simultaneously in more than one state. The states with the highest number of active women vascular surgeons were São Paulo (n = 81), Minas Gerais (n = 46), Bahia (n = 30), and Rio de Janeiro (n = 27).

In response to Question 5 (“How old are you?”), the following age distribution was found: 25-35 years (40.9%, n = 115), 36-45 years (40.6%, n = 114), 46-55 years (13.5%, n = 38), 56-65 years (3.9%, n = 11), and over 65 years (1.1%, n = 3).

Question 6 asked respondents to enter how long (in years) they have worked in their specialty. Results showed that 38.8% (n = 109) of the respondents had worked for ≤5 years; 24.9% (n = 70) for between 6 and 10 years; and 23.8% (n = 67) for between 11 and 20 years; while 12.5% (n = 35) had more than 20 years of experience.

### Specialization

**Figure 1:**
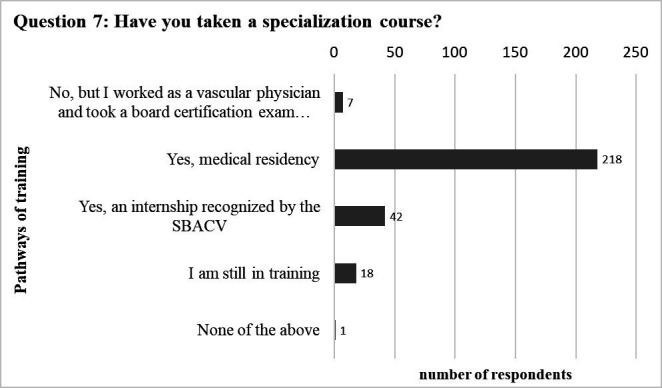
Graph illustrating the training pathway in vascular surgery followed by the interviewees.

Question 7 asked “Have you taken a specialization course?” to collect data on specialty training programs currently adopted in Brazil (Figure 1). This question allowed more than one answer per participant since specialization in endovascular surgery corresponds to an additional year of training, which is optional. Among the respondents, only 2.5% (n = 7) reported that they had not undertaken specific training in vascular surgery. The majority (77.6%, n = 218) had attended a residency program in vascular surgery, 14.9% (n = 42) had attended an SBACV-recognized internship, 18 (6.4%) were still in training, and 1 (0.4%) answered “none of the above.”

Responses to Question 8 (“Do you hold any of the following specialist qualifications?”) served as data on those who had passed SBACV-administered exams (multiple answers were allowed). Overall, 10% (n = 28) of participants reported holding the title of “Specialist in Angiology”; 47% (n = 132) held the title of “Specialist in Vascular Surgery”; 25.3% (n = 71) held the title of “Specialist in Doppler Vascular Sonography”; and 10.3% (n = 29) held the title of “Specialist in Angioradiology and Endovascular Surgery,” while 21.4% (n = 60) had 2 or more of the abovementioned titles and 32.7% (n = 92) reported having no title.

### Economic aspects of professional practice and leadership positions

**Figure 2:**
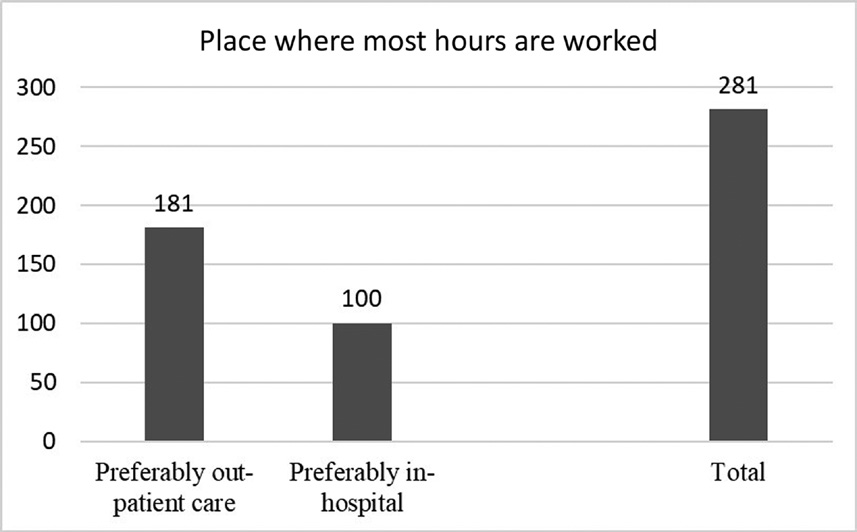
Graph illustrating the distribution of places where most hours are worked by 281 women vascular surgeons in Brazil.

Regarding their position in the workforce, 175 (62.3%) participants reported that they worked simultaneously in the public and private sectors, while 33.5% (n = 94) worked exclusively in the private sector, and only 4.3% (n = 12) worked exclusively in the public sector, according to responses from Question 9. Among women who worked exclusively in the private sector, the majority (79.8%) had at least one title (OR: 0.76, 95% CI: 0.65-0.89; p = 0.001) (Table 1). In response to Question 10 (“You work more hours…”), 64.4% (n = 181) reported that they worked more in the office/clinic, 34.2% (n = 96) worked more in the hospital environment, and 1.4% (n = 4) reported that they distributed their workload proportionally between both environments (Figure 2). There was an association between the number of years working in the specialty and the environment where they spent the highest number of working hours, where the longer the time working in the specialty, the greater the predominance of outpatient care (p = 0.002) (Table 2). Question 11 allowed for more than one answer and asked about the classification of outpatient activity (Supplemental Figure 2). Overall, 37% (n = 104) of participants reported having their own office, 29.9% (n = 84) reported working in a sublet office, and 40.2% (n = 113) reported working in partnership with clinic owners. In response to Question 12 (“Currently, what is your principal field of activity?”), which also allowed for multiple answers, 55.5% (n = 156) of participants indicated venous surgery, 37.7% (n = 106) indicated vascular ultrasound, 43.4% (n = 122) indicated aesthetic phlebology, and 10.3% (n = 29) indicated arterial and endovascular surgery. In addition, 81 participants reported they were unable to specify their main activity, because they performed a mixture of these activities.

**Table 1:** Association between the type of practice (public and/or private) and the board certification of 281 women vascular surgeons in Brazil.

**Variables**	**No specialist title n (%)**	**At least 1 specialist title n (%)**	**p value**	**OR (95%CI)**
Both	64 (36.6)	111 (63.4)	0.076	1.16 (0.98-1.36)
Public Practice	9 (75.0)	3 (25.0)	0.002	2.76 (1.03-7.30)
Private Practice	19 (20.2)	75 (79.8)	0.001	0.76 (0.65-0.89)

Odds ratio (OR) chi-squared test. CI = confidence interval.

**Table 2:** Association between place where most hours are worked and years practicing the specialty among 281 women vascular surgeons in Brazil.

**Variables**	**Up to 5 years n (%)**	**6-10 years n (%)**	**11-20 years n (%)**	**More than 20 years n (%)**	**p value**
Hospital	53 (48.6)	21 (30.0)	17 (25.4)	9 (25.7)	0.004
Clinic	56 (51.4)	49 (70.0)	50 (74.6)	26 (74.3)	0.002

Pearson’s chi-square test.

For Question 13 (“Have you ever held one of the following management roles?”), there were 191 answers (68%) of “No, I have never held a management position,” 60 (21.4%) responses of “Yes, as supervising physician of a service,” and 30 (10.6%) of “Yes, within the SBACV” (Supplemental Figure 3). An analysis stratified by State revealed an association between the State of Minas Gerais and the answer “No, I have never held a management position” reported by participants (OR 2.00, 95% CI 1.04-3.85, p = 0.02). Additionally, for the whole sample, there was an association between participants who reported having their own office and having already held a management position (OR 0.68, 95% CI 0.49-0.96, p = 0.03). A hypothesis that practicing arterial surgery would be related to a higher number of management positions was tested, but no association was found (OR: 0.73, 95% CI: 0.46-1.18; p = 0.24) (Table 3).

**Table 3:** No statistically significant associations were found between the field of practice and leadership positions occupied by 281 women vascular surgeons in Brazil.

**Variables**	**Leadership position**		**p value**	**OR (95% CI)**
	**No n**	**Yes n**		
Arterial surgery	17	12	0.24	0.73 (0.46-1.18)
Venous surgery	107	49	0.91	1.01 (0.72-1.43)
Endovascular surgery	17	12	0.24	0.73 (0.46-1.18)
Vascular sonography	72	34	0.91	0.98 (0.68-1.39)
Esthetic phlebology	90	32	0.084	1.36 (0.95-1.96)
Mixed activities	56	25	0.85	1.03 (0.70-1.52)

Odds ratio (OR) chi-square test. CI = confidence interval.

### Scientific production and impact of gender on career

In response to the question “Have you ever taken part in any of the following academic activities?” (item 14 of the questionnaire), 36.7% (n = 103) of participants marked “Presentation at symposium/congress,” 12.5% (n = 35) marked “Round table speaker at symposium/congress,” 34.9% (n = 98) reported participating in both activities, and 27.8% (n = 78) reported that they had not participated in either activity. This question allowed multiple responses. In response to Question 15, 67.3% (n = 189) reported that they have published scientific work, whereas 32.7% (n = 92) reportedly have had no publications. Finally, Question 16 (“At any point during your career, have you ever felt undervalued or at a disadvantage because you are a woman?”) elicited 172 (61.2%) “Yes” answers and 109 (38.8%) “No” answers.

## DISCUSSION

The present study aimed to describe the demographic and professional profile of women vascular surgeons in Brazil, promoting discussion of strategies for greater integration of women in the specialty.

The results of age distribution analysis corroborate current findings that the mean age of doctors is getting younger.^1^^,^^2^ In addition, almost two-thirds of the professionals evaluated in this study have worked in their specialty for ≤10 years, partly reflecting a greater interest in vascular surgery among women in the last decade.^12^

In Brazil, the most common vascular surgery training route consists of a program lasting (2 + 3 years) or (2 + 2 years), with the first two years corresponding to practice in general surgery and the remaining years in vascular surgery, with or without an optional year in endovascular surgery.^15^ There are no official residency programs that follow (0 + 5 years) with direct access to a vascular surgery specialty. In addition to their basic training, 132 of the 281 respondents reported holding a title of “Specialist in Vascular Surgery” and 60 reported holding 2 or more certifications offered by SBACV, an essential prerequisite for leadership in the Society.^15^ However, only 10.7% reported that they had served in a management position with SBACV.

The low number of respondents in leadership roles follows a worldwide tendency.^16^ Women doctors are less assigned than their male peers to lead specialty societies, editorial boards of journals, academic divisions, and departments of medical education programs and health systems.^16^^-^18 This phenomenon has been observed in several specialties.^19^^,^^20^

Regarding the Brazilian workforce, there is a predominance of women vascular surgeons performing activities that allow for greater work flexibility, such as venous surgery, vascular ultrasonography, and aesthetic phlebology.^12^^,^^21^ Considering that advances in knowledge, production, and the incorporation of new technologies in the field of phlebology require continuous training, women vascular surgeons can serve in new scientific leadership positions.^22^ In addition, since the vast majority of respondents work in the private sector and 37% reported owning their own office, it is understood that there are opportunities for medical entrepreneurship, contributing to entry of women surgeons into the workforce.

The study participants’ scientific involvement is undeniable, considering that 67.3% (n = 189) reported having published scientific work. Understanding and reconciling the limiting factors affecting women's scientific engagement is a process that requires deeper understanding and further clarification.^23^

Despite female interest in surgical specialties showing a tendency to growth, 60% of the participants reported a sense of disadvantage.^24^ A study by Fassiotto et al.^25^ showed that, in general, female medical professors received significantly lower assessment scores than their male counterparts from students, especially in those disciplines in which women are underrepresented, such as surgery. Even during training, there is a discrepancy in recognition of resident physicians’ work so that men are more often awarded than women.^26^^-^28

The present discussion raises the following questions: How can specialty societies act proactively to engage women in the surgical career? What do these institutions have to gain from this?

Currently, it is observed that there is a commitment to expand the medical curriculum, including diversity both as an administrative strategy and as an ethical principle, in order to better prepare graduates to meet the demands of a diverse society. By acting in an inclusive manner, specialty societies will be promoting the LEADS Framework: Lead self, Engage others, Achieve results, Develop coalitions, Systems transformations, thereby corroborating the scientific growth to which they are committed.^29^ We advocate transparency in the selection of representatives, such as direct election of leaders, with clear disclosure of the candidacy processes, as well as the call for volunteering on councils and commissions. In this way, it would be possible to obtain a real sense of the number of men and women interested in participating in this leadership, while also facilitating promotion of diversity in the composition of the groups. By promoting gender diversity, surgical societies would gain through expansion of critical thinking, so necessary in development of specialty guidelines.^5^^,^^11^

In this context, for its 2020/2021 management, the SBACV created a Commission for Strengthening Female Participation, charged with encouraging greater involvement of women vascular surgeons in the association's scientific activities. One of the commission’s proposals is selection of women to participate more actively in panels and debates at specialty congresses and events, giving them recognition and valuation. The commission also proposed creation of the Dr Merisa Garrido Award, to be given to the best paper presented at the Brazilian Congress of Angiology and Vascular Surgery. This tribute to a former president of the society recognizes the memory of someone who strongly contributed to the advance of the specialty. In addition, the initiative will encourage the submission of papers by associate women vascular surgeons, since they will feel represented in the recognition of merit. It is notable that it was also in the 2020 term that 6 of the 23 SBACV regional presidents were female,^30^ the largest female representation in more than 60 years of the institution's existence,^31^ a fact that may have been stimulated by the beginning of our work in 2018, with publication of the preliminary study.

### Limitations

The present study has some limitations that need to be considered. The study questionnaires were sent to women vascular surgeons linked to the SBACV and did not include professionals unaffiliated to the institution. Thus, the results reflect the profile of Brazilian women vascular surgeons associated with the SBACV and, therefore, its interpretations cannot be generalized. Furthermore, questionnaires were sent only once and while this was satisfactory to achieve the minimum sample size needed, statistical validity could have been amplified if the questionnaires had been sent more times. We did not collect data on the male population, so it is not possible to raise comparative hypotheses between genders.

## CONCLUSION

The results of the study suggest that highly qualified women surgeons remain underrepresented in leadership positions throughout Brazil. Promoting greater inclusion and diversity within surgical societies is a necessary strategy to encourage potential female leaders in surgical specialties. Inclusive proposals currently underway at the SBACV are beginning to have an effect. However, their impact deserves to be reported in detail in subsequent studies a few years from now.

## Supplementary Material

Supplementary material accompanies this paper.

Supplemental Table 1 Questionnaire “Women in vascular surgery”, adapted from reference 12.

Supplemental Table 2 Description of sociodemographic variables of 281 women vascular surgeons in Brazil.

Supplemental Figure 1 Distribution of 281 women vascular surgeons by region of Brazil. Values in percentages.

Supplemental Figure 2 Classification of outpatient activity of 281 women vascular surgeons in Brazil

Supplemental Figure 3 Graph illustrating distribution of the participants according to leadership positions.

This material is available as part of the online article from http://www.scielo.br/jvb
