# Supplementary material for: Demographic and professional profile of Brazilian women in vascular surgery: final results
Source: J Vasc Bras. 2021 Aug 13;20:e20210062. doi: 10.1590/1677-5449.210062 (PMC8366404; doi:10.1590/1677-5449.210062)
Supplement: Supplemental Table 1 [file jvb-20-e20210062-suppl01.pdf]

Supplemental Table 1. Questionnaire "Women in vascular surgery", adapted from reference 12.

Question 1. Informed consent form: I declare that I am being invited to participate in the study called "WOMEN IN VASCULAR SURGERY", whose objective is to outline the profile of Brazilian women vascular surgeons, accessing demographic data, characteristics of the specialty in training, economic aspects of the professional practice, scientific engagement, and valuation. My participation in the study will be responding and allowing disclosure of the data found in this questionnaire, being aware that its nature is purely statistical and my personal data will not be evaluated and/or disclosed. I can expect as a benefit of the research the development of a database that serves as a parameter for the design of strategies for better integration of women in the field of Vascular Surgery. The study represents minimal risks to the research subject, since data collection is being processed via the Internet, an environment prone to data exposure. However, in order to minimize the risk of breach of confidentiality, only the principal investigator will have access to the login and password of the collection platform, as well as being solely responsible for checking the completed information and validating the questionnaires. If there are any doubts regarding the study, I can contact the researcher responsible Fernanda Costa Sampaio Silva (main author) by phone (71) 99992-8793 or by email fernandacss81@gmail.com. In case of any type of complaint about this study, I must call CEP Hospital da Bahia or send an email to cep@hospitaldabahia.com.br. I can also personally attend the Hospital da Bahia Study and Research Center, located at Av. Prof Magalhães Neto 1541, Pituba, Salvador-BA.

I agree with the terms of consent:

___Yes ____ No

Question 2. Provide your name and CRM registration number

Question 3. Nationality:

___Brazilian __other (please specify _______)

Question 4. Which state do you practice in?

__Amazonas __Ceará __Pernambuco __ Alagoas __Paraíba

__Sergipe __ Bahia __Tocantins __Goiás __Mato Grosso

__Mato Grosso do Sul __Acre __Espírito Santo __Minas Gerais

__Rio de Janeiro __São Paulo __Paraná __Santa Catarina

__Rio Grande do Sul __Distrito Federal __Amapá __Roraima

__Rondônia __Pará __Rio Grande do Norte __Maranhão __Piauí

Question 5. How old are you?

__25-35 years __36-45 years __46-55 years

__56-65 years __more than 65 years old

Question 6. Time practicing the specialty:

__less than 5 years __6 to 10 years

__11 to 20 years __more than 20 years

Question 7. Have you taken a specialization course?

__No, but I worked as a vascular physician and took a board certification exam afterwards

__Yes, medical residency

__Yes, an internship recognized by the SBACV

__I am still training

__None of the above

Question 8. Do you hold any of the following specialist qualifications?

__Specialist in angiology

__Specialist in vascular surgery

__Certification in vascular ultrasound with Doppler

__Certification in radio angiology or endovascular surgery

__Two or more of the qualifications above

__No specialist qualification

Question 9. You practice...

__Exclusively in the public healthcare system

__Exclusively in private medicine

__Both

Question 10. You work more hours

__Preferably in a consulting room/clinic

__Preferably in a hospital

Question 11. In what type of consulting room/clinic do you work?

__Own

__Sublet

__In partnership with owners/on a percentage basis

__Other (please specify)

Question 12. Currently, what is your principal field of activity?

__Arterial surgery

__Venous surgery

__Endovascular surgery

__Vascular ultrasound

__Esthetic phlebology

__A mixture of activities, unable to specify

Question 13. Have you ever held one of the following management roles?

__Yes, within the SBACV

__Yes, as supervising physician of a service

__No, I have never held a management position

__Yes, a different role (please specify)

Question 14. Have you ever taken part in any of the following academic activities?

__Presentation at symposium/congress

__Round table speaker at symposium/congress

__Both

__Neither of those described above

Question 15. Have you ever had an academic paper published?

__Yes __No

Question 16. At any point during your career have you ever felt undervalued or at a disadvantage because you are a woman?

___Yes __No
