# Supplementary material for: Demographic and professional profile of Brazilian women in vascular surgery: final results
Source: J Vasc Bras. 2021 Aug 13;20:e20210062. doi: 10.1590/1677-5449.210062 (PMC8366404; doi:10.1590/1677-5449.210062)
Supplement: Supplemental Table 2 [file jvb-20-e20210062-suppl02.pdf]

Supplemental Table 2. Description of sociodemographic variables of 281 women vascular surgeons in Brazil.

Variables	n (%)
**Age (Y/O = years old)**	
25 - 35 Y/O	115 (40.9)
36 - 45 Y/O	114 (40.6)
46 - 55 Y/O	38 (13.5)
56 - 65 Y/O	11 (3.9)
More than 65 Y/O	3 (1.1)
**Nationality**	
Brazilian	280 (99.6)
American	1 (0.4)
**State where they practice**	
São Paulo	81 (28.8)
Minas Gerais	46 (16.4)
Bahia	30 (10.7)
Rio de Janeiro	27 (9.6)
Paraná	16 (5.7)
Rio Grande do Sul	15 (5.3)
Distrito Federal	12 (4.3)
Goiás	9 (3.2)
Pernambuco	8 (2.8)
Santa Catarina	7 (2.5)
Mato Grosso do Sul	5 (1.8)
Rio Grande do Norte	4 (1.4)
Espírito Santo	4 (1.4)
Ceará	3 (1.1)

Paraíba	3 (1.1)
Mato Grosso	3 (1.1)
Maranhão	2 (0.7)
Amazonas	1 (0.4)
Acre	1 (0.4)
Rondônia	1 (0.4)
Pará	1 (0.4)
Piauí	1 (0.4)
Sergipe	1 (0.4)
Tocantins	1 (0.4)
Amapá	0 (00.0)
Roraima	0 (00.0)
Alagoas	0 (00.0)
**Time in the specialty**	
Up to 5 years	109 (38.8)
06 - 10 years	70 (24.9)
11 - 20 years	67 (23.8)
More than 20 years	35 (12.5)
