# Supplementary figures and images for: Demographic and professional profile of Brazilian women in vascular surgery: final results
Source: J Vasc Bras. 2021 Aug 13;20:e20210062. doi: 10.1590/1677-5449.210062 (PMC8366404; doi:10.1590/1677-5449.210062)

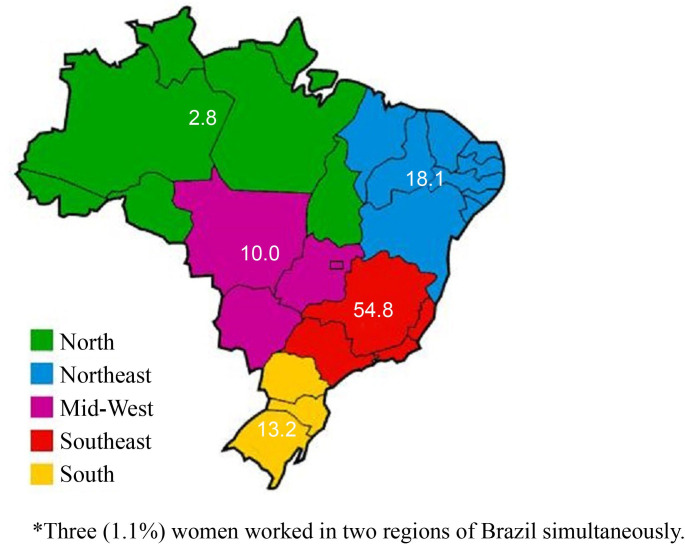

Supplement: Supplemental Figure 1 [file jvb-20-e20210062-suppl03.jpg]

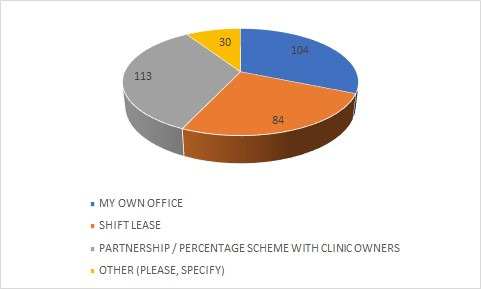

Supplement: Supplemental Figure 2 [file jvb-20-e20210062-suppl04.jpg]

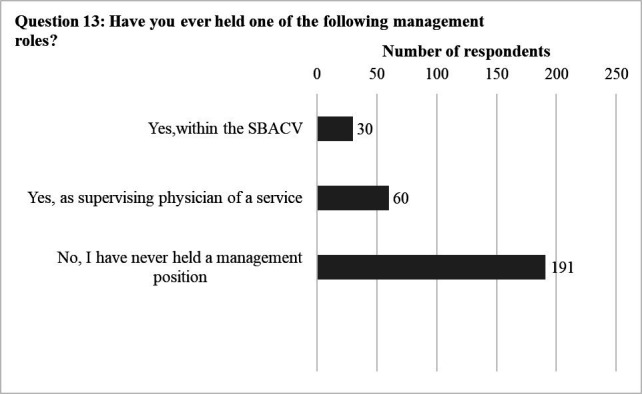

Supplement: Supplemental Figure 3 [file jvb-20-e20210062-suppl05.jpg]
